# Ultra-processed food consumption, socio-demographics and diet quality in Australian adults

**DOI:** 10.1017/S1368980021003967

**Published:** 2022-01

**Authors:** Laura Marchese, Katherine M Livingstone, Julie L Woods, Kate Wingrove, Priscila Machado

**Affiliations:** 1 School of Exercise and Nutrition Sciences, Deakin University, 221 Burwood Hwy, Burwood, VIC 3125, Australia; 2 Institute for Physical Activity and Nutrition (IPAN), School of Exercise and Nutrition Sciences, Deakin University, Geelong, VIC, Australia

**Keywords:** Ultra-processed food, Food processing, Socio-demographic, Diet quality

## Abstract

**Objective:**

To examine how socio-demographic characteristics and diet quality vary with consumption of ultra-processed foods (UPF) in a cross-sectional nationally representative survey of Australian adults.

**Design:**

Using a 24-h recall, this cross-sectional analysis of dietary and socio-demographic data classified food items using the NOVA system, estimated the percentage of total energy contributed by UPFs and assessed diet quality using the Dietary Guideline Index (DGI–2013 total and components). Linear regression models examined associations between socio-demographic characteristics and diet quality with percentage of energy from UPF.

**Setting:**

Australian Health Survey 2011–2013.

**Participants:**

Australian adults aged ≥ 19 years (*n* 8209).

**Results:**

Consumption of UPF was higher among younger adults (aged 19–30 years), adults born in Australia, those experiencing greatest area-level disadvantage, lower levels of education and the second lowest household income quintile. No significant association was found for sex or rurality. A higher percentage of energy from UPF was inversely associated with diet quality and with lower DGI scores related to the variety of nutritious foods, fruits, vegetables, total cereals, meat and poultry, fish, eggs, nuts and seeds, legumes/beans, water and limits on discretionary foods, saturated fat and added sugar.

**Conclusions:**

This research adds to the evidence on dietary inequalities across Australia and how UPF are detrimental to diet quality. The findings can be used to inform interventions to reduce UPF consumption and improve diet quality.

The main causes of pre-mature death and disability in Australia and worldwide are obesity and chronic non-communicable diseases^(1,2)^, with dietary factors being the leading risk for these globally^(3)^. The increase in obesity rates has occurred in parallel with changes in global food systems, which have driven higher consumption of ultra-processed foods (UPF)^(4,5)^. The NOVA system is the food processing classification system most applied in scientific literature^(6)^ and classifies foods into four groups based on the purpose and extent of industrial processing: unprocessed and minimally processed foods, processed culinary ingredients, processed foods and UPF^(7)^. Examples of UPF include instant soups, carbonated soft drinks, mass-produced breads, breakfast ‘cereals’, fast-food dishes, flavoured milk drinks and confectionary. Increased intake of UPF has been associated with an increased risk of obesity and chronic diseases, such as diabetes, CVD, depression and cancer, and mortality^(8–11)^, which are likely to disproportionately affect different socio-demographic groups^(12–18)^.

UPF consumption accounts for 42 % of dietary energy consumed by Australians^(19)^; however, little is known about how UPF consumption is distributed among socio-demographic groups. Individuals with lower socio-economic position such as those from disadvantaged areas or with lower education level have been identified as having poorer diets than those with higher socio-economic position^(20,21)^. Poorer dietary profiles, including greater consumption of energy-dense foods^(22)^ and lower intake of fruit and vegetables^(23,24)^ in those who are more socio-economically disadvantaged, result in poorer overall nutrient intake^(20)^ and higher rates of chronic disease^(25)^.

Identifying how UPF consumption is distributed among socio-demographic characteristics will add to the body of evidence on dietary inequalities across Australia. Additionally, the increasing evidence supporting the use of UPF as a descriptor of unhealthy foods within a dietary pattern has the potential to improve future development of dietary guidelines as well as nutrition policy actions and interventions targeting these inequalities.

The low cost, convenience and high level of marketing amplifies the perceived advantages of UPF over unprocessed or minimally processed foods and freshly prepared meals^(12,26)^. High consumption of UPF may displace the intake of minimally processed foods, leading to nutritionally unbalanced diets^(7)^. In evaluating the effects of consumption of UPF, studies have identified that diets high in these products have the least healthful nutrient profile. However, there is not yet evidence linking UPF consumption to overall diet quality^(7)^. Diet quality is a term used to describe the quality and variety of an individual’s overall diet and is assessed by comparing food and nutrient intakes with dietary guidelines, choices within core food groups or with other international groupings^(27)^. This information is crucial for understanding the prevention of chronic diseases, and also for developing dietary guidelines and health promotion strategies^(28)^.

Despite the evidence that individuals with lower socio-economic position have been identified as having poorer diets than those with higher socio-economic position^(21)^ and diets high in UPF have the least healthful nutrient profile^(7)^, little is known about how UPF consumption is distributed among socio-demographic groups, or their association with diet quality. Using a cross-sectional nationally representative survey of Australian adults, this study aimed to examine whether socio-demographic characteristics and diet quality vary with consumption of UPF.

## Methods

### Study design and participants

This study involved cross-sectional analysis of existing data from the Australian Health Survey 2011–2013, which was conducted by the Australian Bureau of Statistics (ABS)^(29)^. Incorporated within this survey, the National Nutrition and Physical Activity Survey 2011–2012 (NNPAS) was a nationally representative, cross-sectional survey conducted from May 2011 to June 2012^(30)^. A total of 9519 households were recruited using a complex, stratified, multistage probability cluster sampling design^(30)^. Of these households, 12 153 Australians aged 2 years and above provided information on socio-demographic, dietary and lifestyle characteristics^(29)^. This study is reported according to the Strengthening the Reporting of Observational studies in Epidemiology – Nutritional Epidemiology (STROBE-nut) reporting guidelines^(31)^.

**Fig. 1:**
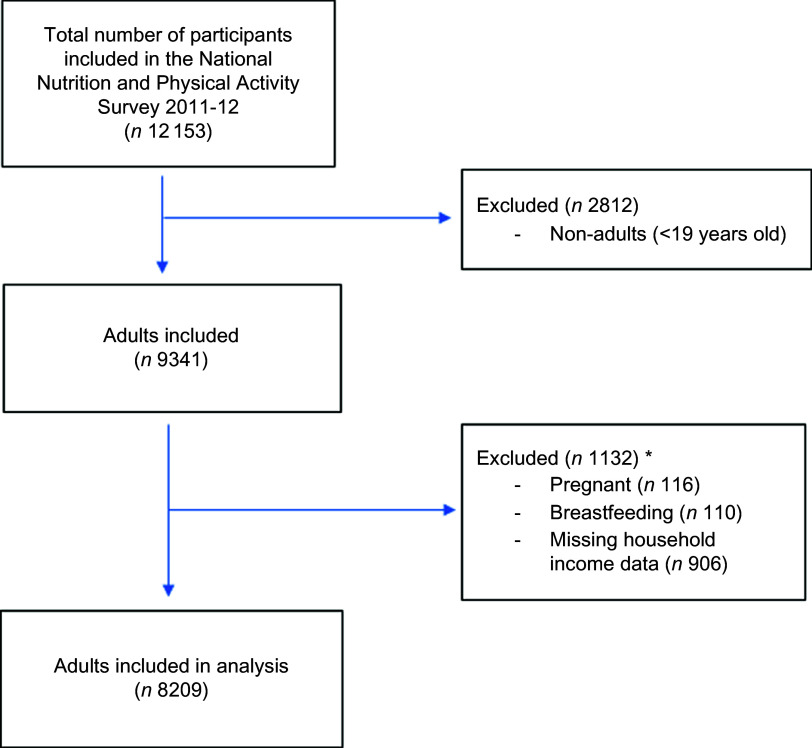
1 Flow diagram of participants included in the analysis of the 2011–12 National Nutrition and Physical Activity Survey. *Categories are not mutually exclusive

For the present study, participants were excluded from analysis if they were (i) less than 19 years of age (*n* 2812), (ii) were pregnant and/or breast-feeding (*n* 226) and (iii) or had missing data for outcomes and exposures (*n* 906 missing data for income) (categories are not mutually exclusive). A total of 8209 participants were included for analyses (Fig. 1).

### Socio-demographic characteristics

Socio-demographic data were collected for all individuals via face-to-face interviews with an ABS trained and experienced interviewer^(30)^. Information was collected for a range of demographic and socio-economic characteristics, including sex, age, country of birth, area-level disadvantage, education, income and rurality^(29)^. For the purpose of this study, sex was categorised as male or female, and age as 19–30, 31–50, 51–70 and 71+ years. Country of birth was assessed using three categories – Australia, main English-speaking country (Canada, Republic of Ireland, New Zealand, South Africa, UK and USA) or other. Area-level disadvantage was calculated using the ABS Index of Relative Socio-economic Disadvantage (SEIFA 2011 – National)^(29)^ and divided into quintiles – lowest 20 % (greatest disadvantage), second quintile, third quintile, fourth quintile, highest 20 % (most advantage). This is a ranking based on the relative socio-economic advantage and disadvantage of the location of the household, derived from variables for income, educational attainment, occupation and economic resources (dwellings with or without motor vehicles). Education was assessed using highest education level completed (both school and non-school) and categorised into tertiles – low (incomplete high-school or less), medium (complete high school or incomplete high school and/or certificate/diploma) and high (tertiary qualification)^(32)^. Income was assessed based on the gross weekly combined equivalised income of all household members aged ≥ 15 years and divided into quintiles of the population – first quintile (≤$398 Australian Dollars (AUD) ($299 United States Dollars (USD)), 20 % lowest income), second quintile ($399–$638 AUD ($300–$480 USD)), third quintile ($639–$958 AUD ($480–$720 USD)), fourth quintile ($959–$1437 AUD ($721–$1080 USD)) and fifth quintile (≥$1438 AUD ($1081 USD), 20 % highest income) (exchange rate correct as of July 5th 2021). Rurality was assessed using the Australian Statistical Geography Standard Remoteness areas categories (2011)^(29)^ and was divided into three categories – major cities of Australia, inner regional Australia and other (outer regional Australia, remote Australia and very remote Australia).

### Dietary intakes

Data on food and beverage consumption were collected using two non-consecutive 24-h dietary recalls^(29)^. Dietary recalls were administered by ABS trained interviewers using the Automated Multiple-Pass Method adapted for use in Australia. The first recall was conducted through a face-to-face interview (*n* 12 153), while the second recall was administered via telephone 8 d or more after the first interview (*n* 7735)^(29,30)^. Energy and nutrient intakes from the recalls were calculated using the Australian Food and Nutrient Database 2011–2013 (AUSNUT 2011–2013), developed by Food Standards Australia New Zealand^(33)^.

### Ultra-processed food consumption

Food and beverages consumed in the NNPAS were previously classified according to the NOVA classification system by Machado *et al.*^(19)^ Briefly, dietary recall data on single food items and the individual ingredients from home-made recipes were classified according to the four NOVA system groups: group 1 – unprocessed or minimally processed foods, for example, fruits, cereals and eggs; group 2 – processed culinary ingredients, for example, salt, plant oils and table sugar; group 3 – processed foods, for example, cheese, processed breads and canned fruit and fish ; group 4 – UPF, for example,instant soups, carbonated soft drinks, mass-produced breads, breakfast ‘cereals’, fast-food dishes, flavoured milk drinks and confectionary (see online supplemental Table 1)^(12)^.

Supplemental Table 1 outlines the twenty-two subgroups which were used to estimate UPF intake^(19)^. Food items were ultimately classified as UPF if they contained ingredients found exclusively in these products, such as food substances of no or rare culinary use, mostly used only in the manufacture of UPF (e.g. protein isolate, invert sugar, hydrogenated oil), or classes of additives with cosmetic functions (e.g. colours, flavours, emulsifiers, artificial sweeteners). More information regarding classifying UPF using NOVA can be found elsewhere^(12)^.

### Diet quality

The present study used the Dietary Guideline Index (DGI) to assess diet quality. The DGI assesses compliance with the 2013 Australian Dietary Guidelines (ADG) for adults, which are the latest dietary guidelines in this country^(34)^. Information on dietary intakes from the 24-h recall were used to score intakes against thireteen dietary components (see online supplemental Table 2). These include seven components which reflect adequate intake of nutritious foods: enjoy a wide variety of nutritious foods; plenty of vegetables; fruit; grain (cereal) foods; lean meat and poultry, fish, eggs, nuts and seeds, and legumes/beans; milk, yoghurt, cheese and/or their alternatives; and drink plenty of water. Another six components reflect moderation or limited intake of foods: limit intake of foods containing saturated fat, added salt, added sugars and alcohol; limit intake of foods high in saturated fat; small allowance of unsaturated oils, fats or spreads; limit intake of foods and drinks containing added salt; limit intake of foods and drinks containing added sugars; and limit alcohol intake. Definitions for each component can be found elsewhere^(32)^.

DGI scores range from 0 to 130, with a higher score indicating better diet quality^(34)^. Each component was scored out of 10 (a score of 0 indicating the dietary guideline was not met), with the exception of grain (cereal) foods, lean meat and poultry, fish, eggs, nuts and seeds, and legumes/beans, drink plenty of water, and limiting intake of foods high in saturated fat which were each comprised of two subcomponents and were scored out of five each. Cut-offs used to obtain the maximum score for each component were tailored to age- and sex-specific food-based recommendations outlined in the ADG^(34)^. Further details on the DGI are available elsewhere^(34)^, with this method used in similar studies^(21,35,36)^.

### Covariates

Covariates were selected based on existing literature and included physical activity, smoking status, BMI and energy misreporting^(37,38)^. Self-reported physical activity and smoking status were collected via questionnaire by the ABS interviewer during the face-to-face interview^(39)^. At the same time, weight (kg) and height (m) measurements were obtained by trained interviewers following standard measurement techniques^(29)^, using digital scales to measure weight and a stadiometer to measure height^(29)^. In this study, physical activity was categorised as having met the recommended 150 min of physical activity in the last week, or not^(29)^. Smoking status was categorised as either current smoker, ex-smoker or never smoked^(29)^. BMI was calculated using Quetelet’s index, using weight (kg) divided by height (m)^2(29)^, and divided into three categories – underweight and normal (BMI < 25 kg/m^2^), overweight (BMI ≥ 25 kg/m^2^ and < 30 kg/m^2^) and obese (BMI ≥ 30 kg/m^2^)^(40)^.

Energy intake misreporting (continuous) was calculated using the ratio of reported total energy intake to predicted total energy expenditure (EI: EE) method^(41)^. Predicted total energy expenditure was calculated using equations suitable for populations with a range of weights and used information on participant age, height and physical activity level^(41)^.

### Statistical analysis

For all analyses, person-specific weights and replicate weights (using jackknife method) were applied to account for selection probability and the effect of complex sampling procedures adopted in the NNPAS^(29)^. Descriptive statistics (mean and standard error) were used to report the distribution of respondents and percentage of energy from UPF (% of total energy intake, continuous) according to socio-demographic characteristics and diet quality (tertiles of DGI score). For the analysis, we used the first 24-h recall, which is suitable for estimating group means^(42,43)^. Socio-demographic variables were categorised as outlined above. The population was categorised into DGI tertiles for descriptive purposes: first tertile – low 13·4–70·5 (mean 60·0), second tertile – medium 70·5–83·9 (mean 77·2) and third tertile – high 84·0–121·0 (mean 93·2). DGI was used as a continuous variable in regression analyses.

Crude (unadjusted) and multivariate (adjusted) linear regression models were used to evaluate the associations between socio-demographic characteristics (dependent variables) and diet quality and its components (dependent variable) with percentage of energy from UPF (independent variable). Multivariate models were adjusted for all socio-demographics and diet quality variables, plus physical activity, smoking status and BMI. The population was further stratified according to quintiles of the percentage of energy from UPF, with the lowest consumers belonging to the first quintile and the highest consumers to the fifth. Intakes of diet quality components were estimated across those quintiles. Linear regression analyses, adjusted for demographics, physical activity, smoking status and BMI, were used to examine associations between the percentage of energy from UPF and the diet quality components.

Sensitivity analyses were carried out by including energy intake misreporting in the multivariate models to evaluate the associations between socio-demographic characteristics and diet quality with percentage of energy from UPF. The EI:EE ratio (continuous) was included as a covariate, an approach used in similar research^(44)^. This method was chosen as previous research suggests that excluding energy misreporters may lead to selection bias due to differences in characteristics of plausible and non-plausible energy reporters^(41,45,46)^.

Weighted analyses were performed using Stata survey module (v16, Stata Corp.). *P*-value was used to evaluate the strength of the associations, with *P* < 0·05 indicative of strong or very strong evidence.

## Results

### Participant characteristics and percentage of energy from ultra-processed foods according to socio-demographic characteristics and diet quality

In 2011–2012, Australian adults (mean age 49·5 years (sd 17·1)) consumed an average of 8416 kJ (se 60·4) per day, 38·8 % (se 0·2) of which were from UPF. The mean diet quality score was 77·2 (se 0·2) (data not shown). Table 1 describes the Australian adult population (distribution and % energy intake from UPF) according to socio-demographic characteristics, tertiles of the diet quality score and covariates (BMI, physical activity and smoking status). In this analysis, 51·7 % of the participants were male, 39·2 % were aged 31–50 years and most participants were born in Australia (69·3 %). Nearly half of the participants had medium-level education (48·9 %), 18·7 % of participants lived in areas with the greatest area level of disadvantage and 18·8 % were from the lowest household income quintile. Majority of participants resided in major cities (70·4 %), 33·4 % of participants were overweight, 50·2 % met physical activity requirements and 50·3 % never smoked (Table 1). Crude percentage energy intake from UPF was highest amongst males (39·7 %), adults aged 19–30 years (44·7 %), born in Australia (41·0 %), experiencing greatest area-level disadvantage (41·6 %), with lowest education level (40·6 %), second lowest household income quintile (41·5 %) and those who reside in inner regional Australia (41·4 %) (Table 1). Crude energy intake from UPF was highest for those in the lowest diet quality score tertile (47·5 %), who are obese (42·0 %), who do not meet physical activity recommendations (40·7 %) and who currently smoke (43·1 %).

**Table 1:** Distribution (%) of the population and mean percentage of energy from ultra-processed foods (% energy intake) according to socio-demographic characteristics and diet quality in Australian adults from the 2011–2012 National Nutrition and Physical Activity Survey (*n* 8209)

Characteristics	Distribution %	se	% energy intake from ultra-processed foods	se
Sex				
Male	51·7	0·4	39·7	0·5
Female	48·3	0·4	38·1	0·4
Age (years)				
19–30	19·9	0·5	44·7	0·9
31–50	39·2	0·4	39·2	0·5
51–70	29·9	0·3	35·1	0·6
71+	11·0	0·2	37·9	0·7
Country of birth				
Australia	69·3	0·8	41·0	0·4
Main English-speaking country	11·8	0·5	38·3	0·9
Other	19·0	0·7	31·7	0·8
Area-level disadvantage*				
First quintile (greater disadvantage)	18·7	1·0	41·6	0·8
Second quintile	20·5	1·0	39·7	0·7
Third quintile	20·8	1·0	39·9	0·8
Fourth quintile	18·9	1·1	38·1	0·9
Fifth quintile (most advantage)	21·0	1·0	35·7	0·7
Education†				
Low	25·6	0·6	40·6	0·7
Medium	48·9	0·8	40·2	0·4
High	25·5	0·7	34·9	0·6
Household income‡				
First quintile (20 % lowest income)	18·8	0·5	37·6	0·6
Second quintile	17·8	0·6	41·5	0·8
Third quintile	20·0	0·6	40·1	0·8
Fourth quintile	21·8	0·6	40·2	0·8
Fifth quintile (20 % highest income)	21·5	0·7	35·6	0·7
Rurality				
Major city of Australia	70·4	0·7	38·1	0·4
Inner regional Australia	19·8	0·9	41·4	0·7
Other	9·8	0·8	40·1	1·1
Diet quality (DGI) score§				
Low (lowest diet quality)	33·3	0·7	47·5	0·6
Medium	33·3	0·6	38·8	0·5
High (highest diet quality)	33·3	0·7	30·5	0·5
BMI‖				
Underweight and normal	31·6	0·7	37·8	0·6
Overweight	33·4	0·7	37·8	0·6
Obese	23·9	0·6	42·0	0·7
Physical activity¶				
Met recommended guidelines	50·2	0·7	37·2	0·4
Did not meet recommended guidelines	49·1	0·7	40·7	0·5
Smoking				
Current smoker	17·7	0·6	43·1	0·9
Ex-smoker	32·0	0·7	37·6	0·5
Never smoked	50·3	0·8	38·3	0·5

DGI, Australian Dietary Guideline Index.

* Calculated using Index of Relative Socio-economic Disadvantage – 2011 – quintiles – national.

† Low (incomplete high school or less), medium (completed high school or incomplete high school and/or certificate/diploma) and high (tertiary qualification).

‡ Combined income of all household members aged ≥ 15 years, divided into quintiles of the population.

§ DGI scores could range between 0 and 130, with a higher score indicating better diet quality – first tertile: low DGI 13 4–70 5 (mean 60 0), second tertile: medium DGI 70 5–83 9 (mean 77 2) and third tertile: high DGI 84 0–121 0 (mean 93 2).

‖ Underweight and normal (BMI < 25 kg/m^2^), overweight (BMI ≥ 25 kg/m^2^ and <30 kg/m^2^), and obese (BMI ≥ 30 kg/m^2^).

¶ Recommended guideline of 150 min of physical activity in the last week; weighted percentages may not add up to 100 for BMI and physical activity due to missing values.

### Associations of ultra-processed food consumption with socio-demographic characteristics and diet quality

Table 2 presents the results from the crude and multivariable linear regression analyses which examined the associations between percentage of total energy from UPF with socio-demographic characteristics and diet quality in Australian adults.

**Table 2:** Associations between percentage of energy from ultra-processed foods (% of total energy) and socio-demographic and diet quality characteristics in Australian adults from the 2011–2012 National Nutrition and Physical Activity Survey (*n* 8209)

Characteristic	% of total energy intake from ultra-processed foods					
	Crude *β*	95 % CI	*P*-value	Adjusted *β*	95 % CI	*P*-value
Sex						
Male	Reference	–	0·017	Reference	–	0·308
Female	−1·7	−3·1, −0·3		−0·8	−2·2, 0·5	
Age (years)						
19–30	Reference	–	<0·001	Reference	–	<0·001
31–50	−5·5	−7·4, −3·6		−4·6	−6·4, −2·9	
51–70	−9·6	−11·6, −7·6		−8·3	−10·3, −6·4	
71+	−6·8	−9·1, −4·5		−5·5	−8·0, −2·9	
Country of birth						
Australia	Reference	–	<0·001	Reference	–	<0·001
Main English-speaking country	−2·8	−4·7, −0·8		−1·2	−2·9, 0·4	
Other	−9·4	−11·2, −7·5		−8·1	−9·8, −6·3	
Area-level disadvantage*						
First quintile (greater disadvantage)	Reference	–	<0·001	Reference	–	0·048
Second quintile	−1·9	−4·0, 0·3		−1·0	−2·8, 0·8	
Third quintile	−1·6	−3·9, 0·5		−0·1	−1·9, 1·7	
Fourth quintile	−3·5	−5·7, −1·3		−0·7	−2·9, 1·4	
Fifth quintile (most advantage)	−5·9	−8·1, −3·7		−2·4	−4·6, −0·1	
Education†						
Low	Reference	–	<0·001	Reference	–	0·005
Medium	−0·4	−1·9, 1·2		−0·8	−2·5, 0·8	
High	−5·7	−7·7, −3·7		−2·3	−4·5, −0·2	
Household income‡						
First quintile (20 % lowest income)	Reference	–	0·016	Reference	–	0·011
Second quintile	4·0	2·1, 5·9		3·4	1·7, 5·1	
Third quintile	2·5	0·5, 4·5		1·9	0·2, 3·5	
Fourth quintile	2·6	0·6, 4·6		2·2	0·3, 4·2	
Fifth quintile (20 % highest income)	−1·9	−3·6, −0·2		−1·2	−3·1, 0·7	
Rurality						
Major city of Australia	Reference	–	0·002	Reference	–	0·904
Inner regional Australia	3·3	1·7, 4·9		0·6	−0·8, 2·1	
Other	2·1	−0·2, 4·3		0·3	−1·8, 2·3	
Diet quality (DGI) score§	−0·5	−0·5, −0·5	<0·001	−0·5	−0·5, −0·4	<0·001

DGI, Australian Dietary Guideline Index.

* Calculated using Index of Relative Socio-economic Disadvantage – 2011 – quintiles – national.

† Low (incomplete high school or less), medium (completed high school or incomplete high school and/or certificate/diploma) and high (tertiary qualification).

‡ Combined income of all household members aged ≥ 15 years, divided into quintiles of the population.

§ DGI scores could range between 0 and 130, with a higher score indicating better diet quality.

Adjusted linear regression analyses were controlled for all the other variables in the table (socio-demographics and diet quality), BMI, physical activity and smoking status.

### Socio-demographic characteristics and consumption of ultra-processed foods

In the crude analysis, all socio-demographic characteristics were strongly associated with percentage of energy from UPF. The multivariable linear regression analysis found strong evidence that all other socio-demographic characteristics, except for sex and rurality, were associated with percentage of energy from UPF. Percentage of energy from UPF was higher among adults aged 19–30 years (43·9 %), and those 51–70 years had the lowest consumption (35·5 %). Adults born in Australia had the highest intake of UPF (40·6 %), while those not born in a main English-speaking country had the lowest intake (32·5 %). Adults experiencing greatest area-level disadvantage had the highest percentage of energy from UPF (39·8 %), while the most advantaged adults by area-level disadvantage had the lowest intake (37·4 %); however, this relationship was not linear across SEIFA quintiles. Percentage of energy from UPF was higher in lower educated adults (39·9 %) and lowest for the higher educated (37·6 %). Adults in the second lowest household income quintile had the highest percentage of energy from UPF (41·1 %) (additional information available in supplemental Table 3).

### Diet quality and consumption of ultra-processed foods

**Fig. 2:**
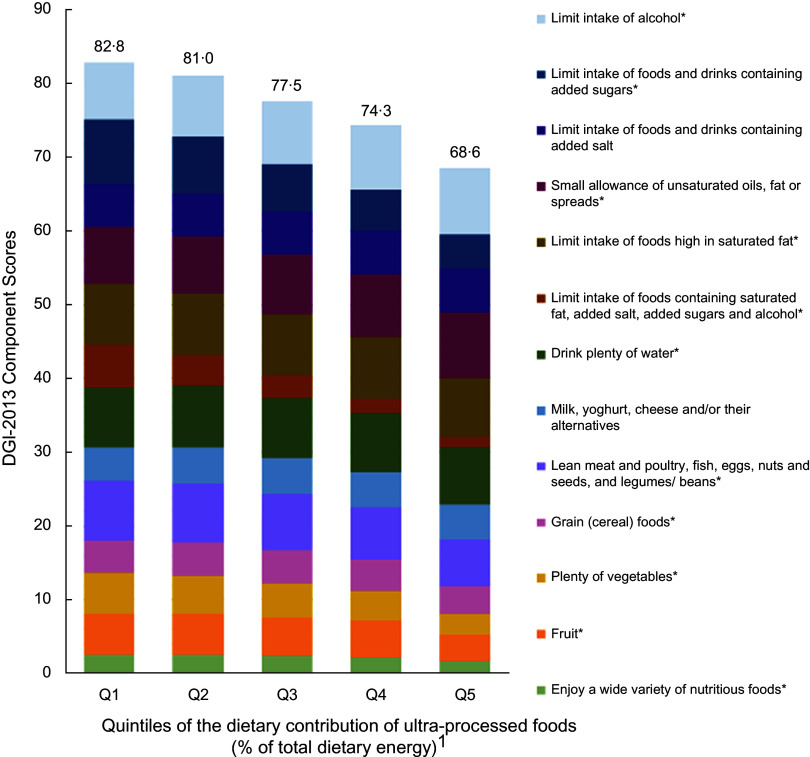
Intakes of diet quality (DGI) components across quintiles of the percentage of energy from ultra-processed foods in Australian adults from the 2011–2012 National Nutrition and Physical Activity Survey (*n* 8209). ^1^Percentage of total energy intake from ultra-processed foods. Mean: Q1 = 11·1 (0–19·3); Q2 = 25·5 (19·3–31·4); Q3 = 36·9 (31·4–42·8); Q4 = 49·8 (42·8–58·2); Q5 = 71·4 (58·3–100). *The association of UPF (continuous) and the diet quality components were significant after adjusting for sex, age, country of birth, area-level disadvantage, education, household income, rurality, physical activity, BMI and smoking status. Values above columns represent the total diet quality (DGI) score which could range between 0 and 130 with a higher score indicating better diet quality. Some components scored inversely (see online supplemental Table 2). DGI, Dietary Guideline Index

Both crude and multivariable models showed that the percentage of energy from UPF was inversely associated with diet quality (adjusted *β* = -0·5, (95 % CI −0·5, −0·4), *P*-value for trend < 0·001) (Table 2). As shown in Fig. 2, higher percentage of energy from UPF was associated with lower diet quality scores for enjoying a wide variety of nutritious foods, fruit, plenty of vegetables, grain (cereal) foods, lean meat and poultry, fish, eggs, nuts and seeds, and legumes/beans, drinking plenty of water, and limiting intake of foods containing saturated fat, added salt, added sugars and alcohol, limiting intake of foods high in saturated fat, and limiting intake of foods and drinks containing added sugars. Both crude and multivariable models showed that the percentage of energy from UPF (continuous) was associated with all diet quality components except for milk, yoghurt, cheese and/or their alternatives and limiting intake of foods and drinks containing added salt (see online supplemental Table 4).

### Sensitivity analyses

Supplemental Table 5 shows the sensitivity analysis results. This included energy misreporting as a covariate in the multivariate linear regression models to evaluate associations of percentage of energy from UPF with socio-demographic characteristics and diet quality. Results show that the inclusion of energy misreporting in the analysis slightly attenuated the associations with the socio-demographics (without affecting the strength of the associations) and did not affect the association with diet quality.

## Discussion

In this cross-sectional analysis of a nationally representative sample of Australian adults, we found higher percentage of energy from UPF among younger people, born in Australia, with greatest area-level disadvantage, low education and second lowest household income. Additionally, the percentage of energy from UPF was inversely associated with diet quality score, and higher percentage of energy from UPF was associated with lower diet quality scores for each of the following features of a healthy diet: enjoying a wide variety of nutritious foods, fruit, plenty of vegetables, grain (cereal) foods, lean meat and poultry, fish, eggs, nuts and seeds, and legumes/beans, drinking plenty of water, and limiting intake of foods containing saturated fat, added salt, added sugars and alcohol, limiting intake of foods high in saturated fat, and limiting intake of foods and drinks containing added sugars. This is the first study in Australia to assess how socio-demographic characteristics and diet quality vary by consumption of UPF. Findings highlight the dietary inequalities among Australian adults associated with UPF consumption and the detrimental effect of their consumption to overall diet quality.

We observed strong evidence for an association between age and UPF consumption, with highest percentage of energy from UPF among the 19–30 age bracket, and lowest percentage of energy from UPF for those 51–70 years of age. These findings are consistent with other national surveys which have all unequivocally identified the youngest adult age bracket studied (ranging between 18 and 39) as having the highest consumption of UPF^(13–15,18,47)^. This age range for young adults is often a time of transition to independent living, undertaking higher education or becoming a parent, and hence changes in dietary behaviours^(48)^. Previous research has identified that young adults (18–25 years old) in this period of transition increase their fast-food consumption, are less likely to plan and organise meals especially during times of stress such as examinations and long work hours, and have a misconception that healthy food is expensive^(48)^. Additionally, young adults are potentially early adopters of new energy-dense foods available in their food environment^(49)^ and are prone to heavy snacking with irregular meal patterns^(48)^. In the present study, those ≥ 71 years of age consumed more UPF than those 51–70 years old. Factors which may be responsible for higher consumption by older adults may include dependence on convenient, ready-to-eat foods and lack of motivation^(50)^. While UPF consumption is highest for younger adults, older age brackets are also susceptible to high consumption, albeit for different reasons.

In the present study, adults in the greatest area-level disadvantage quintile had the highest percentage of energy from UPF, with those most advantaged consuming the least; however, this relationship was not linear across the quintiles. Area-level disadvantage was calculated based on the relative socio-economic advantage and disadvantage of the household. In Australia, income in rural and remote areas is 15–20 % lower than metropolitan areas, and food prices are also higher, which results in rural communities being at higher risk of food insecurity^(51)^. Results from a systematic scoping review conducted with Australian data identified that lower SES groups have overall lower diet quality compared to higher SES groups^(21,25)^. Area-level disadvantage in Australia also affects access to supermarkets with the most advantaged areas having greater access to supermarkets and the less advantaged having closer access to fast-food retailers^(52)^. This leads to those with the greatest area-level disadvantage being more likely to consume diets high in UPF, and also having less access to the wider range of healthy foods found in supermarkets, some of which may be more expensive^(53)^.

We found that those in the second lowest household income quintile consumed the most UPF. Within a country, UPF consumption seems to occur in a socially stratified way, that is, highest consumption initially amongst those with higher income, before shifting to those in lower income groups as a country becomes more affluent^(5)^. How income influences dietary intakes is highly determined by the price of foods^(54)^. Evidence of the cost of ultra-processed *v*. non-UPF diets is not available in Australia. Evidence using other food classification suggests that in Australia healthy diets are cheaper than non-healthy diets^(51,55,56)^ which may help explain why the lowest household income quintile consumed less UPF. Hence, there is likely a number of reasons as to why the second lowest household income quintile consumed the most UPF and a more detailed study of diets at these lower household income levels is required.

To the best of our knowledge, this is the first Australian study to present data associating consumption of UPF with a diet quality score. Previous studies have examined food group and nutrient intakes with UPF consumption and have identified that high UPF consumption was associated with low intakes of fruit and vegetables^(13,17,38,57)^, and altered intakes of nutrients including higher Na, fat, saturated fat and added sugar, and lower intakes of fibre, protein and micronutrients^(7)^. The lower diet quality score for enjoying a wide variety of nutritious foods was expected due to the well-reported nature of UPF displacing intake of minimally or unprocessed foods, regardless of country income^(26)^. These results suggest a shift to a less diversified dietary pattern across countries with high UPF consumption, and a move away from traditional diets^(38)^. In the present study, as UPF consumption increased, the diet quality score for drinking plenty of water fell, which was calculated using total beverage intake and the proportion of water to total beverage intake. Previous studies have identified that soft drinks and sweetened fruit juices are among the UPF contributing most to total energy intake^(14–16,18,38,47,58)^, which is expected due to their hyper-palatability, non-perishable nature, branding and aggressive marketing by transnational and giant corporations makes them extremely appealing.^(12)^ Their flavours, colours, emulsifiers and other additives makes them inherently unhealthy, but also potentially addictive and likely to displace intake of other healthier fluids^(12,59–61)^. Hence, the observed reduction for drinking plenty of water as UPF consumption increases is expected as the consumption of water may be replaced by ultra-processed beverages, such as soft drinks and fruit drinks.

The original evidence from this study can be used to inform policy and practice. The results demonstrate that higher UPF consumption was associated with lower diet quality, which implies that interventions to reduce the consumption of UPF would also improve diet quality. A range of policy interventions could be used to reduce UPF consumption and hence improve diet quality by creating healthier food environments. At the federal government level, policy options include restricting advertising of UPF, introducing taxes on UPF and using food labelling interventions to ensure UPFs can be easily identified by consumers^(12)^. At the state government level, policy interventions can alter product placement and reduce the proportion of UPF for sale in schools and hospitals to encourage healthier food choices^(62,63)^.

The findings from this study have implications for future research. Specifically, future research should identify the particular UPF with the highest consumption among each socio-demographic characteristic as well as any associations with health outcomes such as obesity and CVD, so that appropriate, targeted action can be taken. Additionally, this study could be repeated for younger population groups including, infants, children and adolescents. Further research is also required to identify the policies and programmes which will be the most successful at reducing consumption of UPF and hence improve diet quality in the Australian context^(64)^. In light of the evidence that consumption of UPF relates to overall population diet quality, advances in nutrition research could include the development of a dietary pattern index based on the level of processing using the NOVA system. With this approach, researchers could measure compliance with a specific healthy dietary pattern based on scores reflecting food processing and NOVA subgroups, including its association with a range of potential health outcomes. Continued research in this area is therefore warranted in order to improve our understanding and identification of important determinants of consumption of UPF in Australia and reduce their overall impact.

A strength of the study was the use of the NOVA system, which is the most widely used food processing classification system in research and policy^(6)^ and is valuable for comparing studies using similar, objective and clear methodology to classify foods^(65)^. A strength of using a composite diet quality score such as the DGI-2013 is that it captured intakes of food groups relative to the ADG and hence can provide evidence regarding how UPF intake is affecting the food group intakes in line with current Australian recommendations. A further strength was the inclusion of energy misreporting as a sensitivity analysis. We expected that people who under-reported energy intake would report consuming less UPF, thus introducing social desirability bias (41); however, the results show minimal impact of energy misreporting in the assessment of UPF consumption and hence was not relevant.

Certain limitations of this study include the use of only the first day of 24-h dietary recall, as the second recall was only completed by 64 % of the population^(29)^ and we did not want to introduce potential selection bias. A potential limitation is that the 24-h recall instrument and the food composition tables were not designed for evaluating food based on the level of processing^(19)^. Hence, some items may have been misclassified. However, this was minimised by using a systematic method to classify foods which was revised by experts in order to reduce the chance of misclassification^(19)^. Another potential limitation refers to the calculation of energy misreporting, as it assumed subjects had low physical activity levels, which is unlikely for the whole population. A particular limitation of the study is the age of the NNPAS data, which is almost a decade old^(30)^. Although the landscape of UPF available in Australia is likely to have changed since these data were collected in 2011, these data remain the most detailed nationally representative data on dietary intake available in Australia. The data collected in this analysis will be important for examining trends in UPF intake over time in Australia as any later publications using nationally representative data will need to know what previous intakes were so as to examine how the landscape has changed.

## Conclusions

This study showed that Australian adults aged 19–30 years old, those born in Australia, with greatest area-level disadvantage, low education and second lowest household income quintile had higher intakes of UPF. There was no evidence that sex and rurality were associated with UPF consumption. Additionally, percentage of energy from UPF was inversely associated with diet quality, and higher percentage of energy from UPF was associated with lower DGI scores for food variety, fruit, vegetable, cereal (total), meat and alternatives (total), fluid intake (total), and limiting discretionary, saturated fat and extra sugar. This research adds to the body of evidence on dietary inequalities across Australia and how UPF consumption is detrimental to overall diet quality. The findings can be used to inform interventions to reduce consumption of UPF and improve diet quality.

## Supplementary material

For supplementary material accompanying this paper visit https://doi.org/10.1017/S1368980021003967. click here to view supplementary material
