# Supplementary material for: Ultra-processed food consumption, socio-demographics and diet quality in Australian adults
Source: Public Health Nutr. 2021 Sep 13;25(1):94–104. doi: 10.1017/S1368980021003967 (PMC8825971; doi:10.1017/S1368980021003967)
Supplement: Supplementary file 1 [file S1368980021003967sup001.docx]

##### **Supplementary Material**

##### Supplementary Table 1: Groups and subgroups of NOVA food classification system applied to the AUSNUT 2011-13 and NNPAS 2011-12

| NOVA Group |  |
| --- | --- |
| 1 | **Unprocessed or minimally processed foods** |
|  | Milk and plain yoghurt |
|  | Fruits |
|  | Potatoes and other tubers and roots |
|  | Red meat |
|  | Poultry |
|  | Cereals |
|  | Pasta |
|  | Vegetables |
|  | Eggs |
|  | Nuts and seeds |
|  | Fish |
|  | Legumes |
|  | Other unprocessed or minimally processed foods ^a^ |
|  | Freshly squeezed juices |
| 2 | **Processed culinary ingredients** |
|  | Salt |
|  | Table sugar |
|  | Animal fats |
|  | Plant oil |
|  | Other processed culinary ingredients ^b^ |
| 3 | **Processed foods** |
|  | Beer and wine |
|  | Cheese |
|  | Vegetables and other plant foods preserved in brine |
|  | Processed breads |
|  | Bacon and other salted, smoked or canned meat or fish |
|  | Other processed foods ^c^ |
| 4 | **Ultra-processed foods** |
|  | Mass-produced packaged breads |
|  | Packaged ready meals |
|  | Breakfast cereals |
|  | Sausage and other reconstituted meat products |
|  | Confectionary |
|  | Ice cream, ice pops and frozen yogurts |
|  | Biscuits |
|  | Pastries, buns, and cakes |
|  | Industrial French fries |
|  | Margarine and other spreads |
|  | Milk-based drinks |
|  | Sauces, dressing and gravies |
|  | Packaged salty snacks |
|  | Frozen pizza |
|  | Industrial desserts |
|  | Instant and canned soups |
|  | Soft drinks, carbonated |
|  | Fruit drinks and iced teas |
|  | Alcoholic distilled drinks |
|  | Ultra-processed cheese |
|  | Other ultra-processed foods ^d^ |
|  | Fast food dishes |

^a^ Including non-presweetened, non-flavoured coffee and tea; coconut water; ^b^ Including vinegar; baking powder and baking soda; ^c^ Including salted or sugared nuts and seeds; peanut, sesame, cashew; ^d^ Including soya products such as meatless patties and fish sticks; baby food and baby formula. Reproduced with permission ^(1)^

##### Supplementary Table 2: Components and scoring methods of the revised Dietary Guideline Index (DGI-2013)

| Dietary Guideline | Component and Description | Criteria for Maximum Score ^1^ | Criteria for Minimum Score | Maximum Score |
| --- | --- | --- | --- | --- |
| Guidelines for adequate intake | | | | |
| 1. Enjoy a wide variety of nutritious foods | Food variety ^2^: proportion of food from each of the 5 core food groups eaten at least one serve per week | 100% | 0% | 10 |
| 2. Plenty of vegetables | Total vegetable intake: servings of vegetables per day | 19–50 y: M ≥ 6, F ≥ 5 | 0 | 10 |
|  |  | 51–70 y: M ≥ 5.5, F ≥ 5 |  |  |
|  |  | > 70 y: M ≥ 5, F ≥ 5 |  |  |
| 3. Fruit | Total fruit intake: servings of fruit per day | ≥2 | 0 | 10 |
| 4. Grain (cereal) foods | Total cereal intake: servings of grains per day | 19–50 y: M ≥ 6, F ≥ 6 | 0 | 5 |
|  |  | 51–70 y: M ≥ 6, F ≥ 4 |  |  |
|  |  | >70 y: M ≥ 4.5, F ≥ 3 |  |  |
|  | Mostly wholegrain or high fibre cereals: Type of bread usually consumed | Wholemeal bread | White bread | 5 |
| 5. Lean meat and poultry, fish, eggs, nuts and seeds, and legumes/beans | Total meat and alternative: servings per day | 19–50 y: M ≥ 3, F ≥ 2.5 | 0 | 5 |
|  |  | 51–70 y: M ≥ 2.5, F ≥ 2 |  |  |
|  |  | >70 y: M ≥ 2.5, F ≥ 2 |  |  |
|  | Lean meat: proportion of lean meats and alternatives to total meat and alternatives per day | 100% | 0% | 5 |
| 6. Milk, yoghurt, cheese and/or their alternatives ^3^ | Total dairy and alternative: servings per day | 19–50 y: M ≥ 2.5, F ≥ 2.5 | 0 | 10 |
|  |  | 51–70 y: M ≥ 2.5, F ≥ 4 |  |  |
|  |  | >70 y: M ≥ 3.5, F ≥ 4 |  |  |
| 7. Drink plenty of water | Total beverage intake ^4^: servings per day | M ≥ 10; F ≥ 8 | 0 | 5 |
|  | Water ^5^: proportion of water to total beverage intake per day | ≥50% | 0% | 5 |
| Guidelines to limit or moderate intake | | | | |
| 8. Limit intake of foods containing saturated fat, added salt, added sugars and alcohol | Limit discretionary foods | M ≤ 3; F ≤ 2.5 | M > 3; F > 2.5 | 10 |
| 9. Limit intake of foods high in saturated fat | Trim meat: trimming fat from meat | Usually | Never or rarely | 5 |
|  | Choose reduced-fat milk: type of milk usually consumed | Skim, low or reduced fat milk | Whole milk | 5 |
| 10. Small allowance of unsaturated oils, fats or spreads | Unsaturated spreads and oils: servings per day | 19–50 y: M ≤ 4, F ≤ 2 | M > 4; F > 2 | 10 |
|  |  | 51–70 y: M ≤ 4, F ≤ 2 |  |  |
|  |  | >70 y: M ≤ 2, F ≤ 2 |  |  |
| 11. Limit intake of foods and drinks containing added salt | Salt use: salt added during cooking | Never or rarely | Usually | 5 |
|  | Salt use: salt added during the meal | Never or rarely | Usually | 5 |
| 12. Limit intake of foods and drinks containing added sugars | Limit extra sugar ^6^: servings per day | M ≤ 1.5; F ≤ 1.25 | M > 1.5; F > 1.25 | 10 |
| 13. If you choose to drink alcohol, limit intake | Limit alcohol: servings per day | ≤2 | >2 | 10 |

^1^: Criteria for maximum scores were derived from the Australian Dietary Guidelines ^(2)^ unless otherwise noted; y: years; M: Male; F: Female; ^2^: Food variety was measured and scored using a similar method to the Recommended Food Score ^(3)^; ^3^: Choosing reduced fat dairy is captured in the “Limit intake of foods high in saturated fat” component; ^4^: The Eat for Health Australian Dietary Guidelines do not have specific recommendations for beverage consumption and recommended the guidelines found in the Nutrient Reference Values for Australia and New Zealand ^(4)^; ^5^: The proportion of water to total beverage intake was derived from US beverage guidelines ^(5)^; ^6^: Since added sugar intake is not recommended there are no cut-off values for the number of recommended servings, instead half of the maximum discretionary food cut-off were used consistent with the original DGI. ^(6)^ Reproduced with permission. ^(7)^

##### Supplementary Table 3: Adjusted ^¥^ mean dietary share of ultra-processed foods (% energy intake) according to socio-demographic characteristics and diet quality in Australian adults from the 2011-12 National Nutrition and Physical Activity Survey (n = 8,209).

| **Characteristics** | | **% energy intake from ultra-processed foods (SE)** |
| --- | --- | --- |
|  |  |  |
| **Sex** | |  |
|  | Male | 39.3 (0.5) |
|  | Female | 38.5 (0.4) |
| **Age (years)** | |  |
|  | 19-30 | 43.9 (0.9) |
|  | 31-50 | 39.2 (0.4) |
|  | 51-70 | 35.5 (0.5) |
|  | 71+ | 38.4 (0.8) |
| **Country of Birth** | |  |
|  | Australia | 40.6 (0.4) |
|  | Main English-Speaking Country | 39.4 (0.7) |
|  | Other | 32.5 (0.7) |
| **Area Level Disadvantage ^a^** | |  |
|  | First quintile (greater disadvantage) | 39.8 (0.8) |
|  | Second quintile | 38.8 (0.6) |
|  | Third quintile | 39.7 (0.7) |
|  | Fourth quintile | 39.1 (0.8) |
|  | Fifth quintile (most advantage) | 37.4 (0.7) |
| **Education ^b^** | |  |
|  | Low | 39.9 (0.7) |
|  | Medium | 39.1 (0.4) |
|  | High | 37.6 (0.6) |
| **Household income ^c^** | |  |
|  | First quintile (20% lowest income) | 37.7 (0.6) |
|  | Second quintile | 41.1 (0.8) |
|  | Third quintile | 39.6 (0.6) |
|  | Fourth quintile | 40.0 (0.7) |
|  | Fifth quintile (20% highest income) | 36.5 (0.7) |
| **Rurality** | |  |
|  | Major city of Australia | 38.8 (0.3) |
|  | Inner Regional Australia | 39.4 (0.6) |
|  | Other | 39.0 (1.0) |
| **Diet quality (DGI) score ^d^** | |  |
|  | Low (lowest diet quality) | 46.4 (0.6) |
|  | Medium | 39.2 (0.5) |
|  | High (highest diet quality) | 31.2 (0.5) |

Note: SE, standard error; DGI, Australian Dietary Guideline Index; ^¥^ Adjusted means derived from linear regression analyses controlled for all the other variables in the table (socio-demographics and diet quality), and BMI, physical activity, and smoking status; ^a^ calculated using Index of Relative Socio-economic Disadvantage - 2011 - Quintiles – National; ^b^ low (incomplete high school or less), medium (completed high school or incomplete high school and/or certificate/diploma), and high (tertiary qualification); ^c^ combined income of all household members aged ≥15 years, divided into quintiles of the population; ^d^ DGI scores could range between 0 and 130, with a higher score indicating better diet quality – low DGI 13.4-70.5 (mean 60.0), medium DGI 70.5-83.9 (mean 77.2), high DGI 84.0-121.0 (mean 93.2).

##### Supplementary Table 4: Analysis of the associations between dietary share of ultra-processed foods (% of total energy) and diet quality (DGI) components in Australian adults from the 2011-12 National Nutrition and Physical Activity Survey (n = 8,209).

|  | Dietary contribution of ultra-processed foods (% of total energy intake) | | | | | |
| --- | --- | --- | --- | --- | --- | --- |
| DGI component | **Crude β** | **95% CI** | **p-value** | **Adjusted β** | **95% CI** | **p-value** |
| Food variety | -3.9 | -4.3 to -3.4 | <0.001 | -3.5 | -3.9 to -3.0 | <0.001 |
| Fruit | -0.9 | -1.0 to -0.7 | <0.001 | -0.7 | -0.8 to -0.5 | <0.001 |
| Vegetables | -1.8 | -2.0 to -1.6 | <0.001 | -1.7 | -1.8 to -1.5 | <0.001 |
| Cereal (total) | -0.6 | -0.8 to -0.3 | <0.001 | -0.4 | -0.6 to -0.1 | 0.003 |
| Meat and alternatives (total) | -2.0 | -2.3 to -1.8 | <0.001 | -1.9 | -2.1 to -1.7 | <0.001 |
| Total dairy and alternatives | 0.1 | -0.1 to 0.3 | 0.296 | -0.1 | -0.3 to 0.1 | 0.525 |
| Fluid intake (total) | -0.7 | -1.0 to -0.4 | <0.001 | -0.7 | -1.0 to -0.4 | <0.001 |
| Limit discretionary foods | -1.5 | -1.6 to -1.4 | <0.001 | -1.4 | -1.5 to -1.3 | <0.001 |
| Limit saturated fat (total) | -0.5 | -0.8 to -0.3 | <0.001 | -0.4 | -0.6 to -0.2 | 0.002 |
| Moderate unsaturated-fat | 0.7 | 0.5 to 0.9 | <0.001 | 0.6 | 0.4 to 0.7 | <0.001 |
| Limit added salt (total) | 0.1 | -0.2 to 0.3 | 0.544 | 0.1 | -0.1 to 0.3 | 0.502 |
| Limit extra sugar | -1.4 | -1.5 to -1.3 | <0.001 | -1.3 | -1.4 to -1.2 | <0.001 |
| Limit alcohol | 0.7 | 0.5 to 0.8 | <0.001 | 0.8 | 0.6 to 1.0 | <0.001 |

Note: CI, Confidence Interval; DGI, Australian Dietary Guideline Index; ; ^¥^ Adjusted linear regression analyses were controlled for sex, age, country of birth, area level disadvantage, education, household income, rurality, physical activity, BMI and smoking status; area level disadvantage calculated using Index of Relative Socio-economic Disadvantage - 2011 - Quintiles – National; education defined as low (incomplete high school or less), medium (completed high school or incomplete high school and/or certificate/diploma), and high (tertiary qualification); household income calculated as combined income of all household members aged ≥15 years, divided into quintiles of the population

##### Supplementary Table 5: Sensitivity analysis of the associations between dietary share of ultra-processed foods (% of total energy) and socio-demographic and diet quality characteristics with additional adjustment for energy intake misreporting in Australian adults from the 2011-12 National Nutrition and Physical Activity Survey (n = 8,209).

| Characteristic | | Dietary contribution of ultra-processed foods (% of total energy intake) | | |
| --- | --- | --- | --- | --- |
|  |  | **Adjusted β** | **95% CI** | **p-value** |
| Sex | |  |  |  |
|  | Male | Reference | - | 0.862 |
|  | Female | -0.4 | -1.9 to 1.1 |  |
| Age | |  |  |  |
|  | 19-30 | Reference | - | <0.001 |
|  | 31-50 | -4.2 | -6.1 to -2.4 |  |
|  | 51-70 | -8.1 | -10.2 to -6.1 |  |
|  | 71+ | -5.2 | -7.8 to -2.6 |  |
| Country of Birth | |  |  |  |
|  | Australia | Reference | - | <0.001 |
|  | Main English-Speaking Country | -1.2 | -2.8 to 0.4 |  |
|  | Other | -7.9 | -9.8 to -6.0 |  |
| Area Level Disadvantage ^a^ | |  |  |  |
|  | First quintile (greater disadvantage) | Reference | - | 0.017 |
|  | Second quintile | -0.6 | -2.4 to 1.3 |  |
|  | Third quintile | -0.3 | -2.2 to 1.7 |  |
|  | Fourth quintile | -1.3 | -3.5 to 1.0 |  |
|  | Fifth quintile (most advantage) | -2.6 | -5.0 to -0.3 |  |
| Education ^b^ | |  |  |  |
|  | Low | Reference | - | 0.027 |
|  | Medium | -0.5 | -2.3 to 1.2 |  |
|  | High | -1.8 | -4.0 to 0.4 |  |
| Household income ^c^ | |  |  |  |
|  | First quintile (20% lowest income) | Reference | - | 0.017 |
|  | Second quintile | 4.1 | 2.0 to 6.1 |  |
|  | Third quintile | 2.5 | 0.8 to 4.3 |  |
|  | Fourth quintile | 2.6 | 0.5 to 4.7 |  |
|  | Fifth quintile (20% highest income) | -0.7 | -2.8 to 1.4 |  |
| Rurality | |  |  |  |
|  | Major city of Australia | Reference | - | 0.843 |
|  | Inner Regional Australia | 1.1 | -0.5 to 2.6 |  |
|  | Other | -0.4 | -2.4 to 1.6 |  |
| Diet quality (DGI) score ^d^ | | -0.5 | -0.5 to -0.4 | <0.001 |

CI, confidence interval; ^a^ calculated using Index of Relative Socio-economic Disadvantage - 2011 - Quintiles – National; ^b^ low (incomplete high school or less), medium (completed high school or incomplete high school and/or certificate/diploma), and high (tertiary qualification); ^c^ combined income of all household members aged ≥15 years, divided into quintiles of the population; ^d^ DGI scores could range between 0 and 130, with a higher score indicating better diet quality; ^¥^Adjusted linear regression analyses were controlled for all the other variables in the table (socio-demographics and diet quality), and BMI, physical activity, smoking status, and energy intake misreporting.

**References:**

1. Machado PP, Steele EM, Levy RB*, et al.* (2019) Ultra-processed foods and recommended intake levels of nutrients linked to non-communicable diseases in Australia: evidence from a nationally representative cross-sectional study. *BMJ Open* **9**, (8), e029544.

2. National Health and Medical Research Council. (2013) Australian Dietary Guidelines. Canberra: National Health and Medical Research Council.

3. Kant AK, Schatzkin A, Graubard BI*, et al.* (2000) A prospective study of diet quality and mortality in women. *JAMA* **283**, (16), 2109-15.

4. National Health and Medical Research Council. (2006) Nutrient reference values for Australia and New Zealand. Canberra: National Health and Medical Research Council.

5. Popkin BM, Armstrong LE, Bray GM*, et al.* (2006) A new proposed guidance system for beverage consumption in the United States. *Am J Clin Nutr* **83**, (3), 529-42.

6. McNaughton SA, Ball K, Crawford D*, et al.* (2008) An index of diet and eating patterns is a valid measure of diet quality in an Australian population. *J Nutr* **138**, (1), 86-93.

7. Livingstone KM, Olstad DL, Leech RM*, et al.* (2017) Socioeconomic inequities in diet quality and nutrient intakes among Australian adults: findings from a nationally representative cross-sectional study. *Nutrients* **9**, (10).
